# Glycolipids Recognized by A2B5 Antibody Promote Proliferation, Migration, and Clonogenicity in Glioblastoma Cells

**DOI:** 10.3390/cancers11091267

**Published:** 2019-08-28

**Authors:** Nathalie Baeza-Kallee, Raphaël Bergès, Aurélie Soubéran, Carole Colin, Emilie Denicolaï, Romain Appay, Aurélie Tchoghandjian, Dominique Figarella-Branger

**Affiliations:** 1 Aix Marseille University, CNRS, INP, Inst Neurophysiopathol, Marseille, France; 2 Service d’Anatomie Pathologique et de Neuropathologie, Hôpital de la Timone, AP-HM, Marseille, France

**Keywords:** A2B5, ST8SIA3, glioblastoma, cancer stem cell, tumorigenicity

## Abstract

A2B5+ cells isolated from human glioblastomas exhibit cancer stem cell properties. The A2B5 epitope belongs to the sialoganglioside family and is synthetized by the ST8 alpha-N-acetyl-neuraminidase α-2,8-sialyltransferase 3 (ST8SIA3) enzyme. Glycolipids represent attractive targets for solid tumors; therefore, the aim of this study was to decipher A2B5 function in glioblastomas. To this end, we developed cell lines expressing various levels of A2B5 either by genetically manipulating ST8SIA3 or by using neuraminidase. The overexpression of ST8SIA3 in low-A2B5-expressing cells resulted in a dramatic increase of A2B5 immunoreactivity. ST8SIA3 overexpression increased cell proliferation, migration, and clonogenicity in vitro and tumor growth when cells were intracranially grafted. Conversely, lentiviral ST8SIA3 inactivation in low-A2B5-expressing cells resulted in reduced proliferation, migration, and clonogenicity in vitro and extended mouse survival. Furthermore, in the shST8SIA3 cells, we found an active apoptotic phenotype. In high-A2B5-expressing cancer stem cells, lentiviral delivery of shST8SIA3 stopped cell growth. Neuraminidase treatment, which modifies the A2B5 epitope, impaired cell survival, proliferation, self-renewal, and migration. Our findings prove the crucial role of the A2B5 epitope in the promotion of proliferation, migration, clonogenicity, and tumorigenesis, pointing at A2B5 as an attractive therapeutic target for glioblastomas.

## 1. Introduction

Glioblastomas (GBM) are the most malignant primary intracranial tumors in adults and are mainly characterized by necrosis, high proliferation, invasion, and neoangiogenesis [1]. Furthermore, GBM are composed of several cell types including tumor-initiating cells or cancer stem cells (CSCs), which are resistant to cell death and drive relapse. To increase treatment efficiency, targeting CSCs remains a major therapeutic challenge. 

Gangliosides are sialic-acid-containing glycosphingolipids that are most abundant in the nervous system. During brain development, the levels and patterns of gangliosides shift from simple gangliosides such as GM3 and GD3 to more complex ones such as GM1, GD1a, GD1b, and GT1B (for a review, see [2]). More than 20 years ago, some studies reported that in contrast to normal brain, gliomas express large amounts of GD2 and GD3 [3,4]. Moreover, treatment of glioma cells with the anti-ganglioside A2B5 monoclonal antibody reduced their migration in vitro [4]. It has been shown that GBM selectively express O-acetyl GD2 ganglioside [5]. More recently, the direct role of GD2 and GD3 expression in glioma invasion, motility, and tumor growth has been reported [6,7].

A few years ago, we demonstrated in various glioma subtypes the presence of glial precursor cells expressing c-series gangliosides recognized by the A2B5 monoclonal antibody [8]. The A2B5 monoclonal antibody, originally prepared against chicken embryo retina cells [9], mainly recognizes trisialoganglioside GT3 at the surface of the cells [10]. These antigens, widely expressed during early brain development [2,11], are also known to characterize a small fraction of human adult subcortical white matter cells that have stem cell properties [12]. Several ganglioside markers including SSEA3, SSEA4, GD2, and GD3 have been identified in GBM CSCs [13]. Moreover, we have shown that the A2B5 epitope is another GBM CSC surface marker. In GBM, A2B5+ cells are able to migrate, divide, and differentiate into oligodendroglial and type 1 and 2 astroglial cells [8]. In agreement with other studies, we showed that only A2B5+ cells and not A2B5- cells display a high proliferation index, have the potential to generate primary and secondary spheres, and to develop tumors after orthotopic injection in *nude* mice independently of CD133 [14,15,16].

Altogether, these studies point out that gangliosides represent attractive GBM therapeutic targets. Gangliosides expressed at the cell surface are key regulators of cell recognition and signaling. It is therefore not surprising that they play a pleiotropic role in development and cancer. Gangliosides function in two distinct modes: *cis* and *trans* [17]. In the *cis* mode, gangliosides associate laterally with other membrane molecules, including receptors and ion channels, to modulate their activities. As an example, it has been shown that the ganglioside GD2 enhanced proliferation of breast cancer cells through the constitutive activation of the c-MET receptor [18]. In the *trans* mode, gangliosides—which extend into the extracellular space—interact with complementary glycan-binding proteins, thereby modifying cell-cell or cell-extracellular matrix interactions. Of particular interest is the negative influence of cell surface sialosides on immune cell function by interacting with the immune-inhibitory sialic-acid-binding immunoglobulin-like lectin (Siglec) family (reviewed in [19,20]). Therefore, cell surface sialosides are exploited by tumors to evade both innate and adaptative immune destruction.

The aim of this study was to uncover which properties are conferred to GBM tumor cells by the expression of the A2B5 epitope. To achieve this goal, we manipulated A2B5 expression by genetically modifying its synthesis. It is known that A2B5 results in the addition of a third sialic acid on its precursor GD3 by the golgian ganglioside-specific ST8 alpha-N-acetyl-neuraminide α-2,8-sialyltransferase 3 (ST8SIA3). We overexpressed or suppressed the ST8SIA3 enzyme in GBM cell lines with different basal levels of A2B5, then studied their proliferation, migration, and clonogenicity in vitro and tumorigenesis ability in vivo. Because shST8SIA3 delivery in A2B5-high-expressing cells prevents continuous cell growth, as an alternative we used neuraminidase (sialidase) to cleave the sialic acid residues in α-2,8 to down-regulate A2B5 immunoreactivity. In these models we demonstrated that the A2B5 level is positively correlated with cell proliferation, migration, clonogenicity, and tumorigenicity. Therefore, the glycolipids recognized by the A2B5 antibody are attractive targets for GBM therapy.

## 2. Results

### 2.1. Expression of ST8SIA3 Drives A2B5 Immunoreactivity

**Figure 1:**
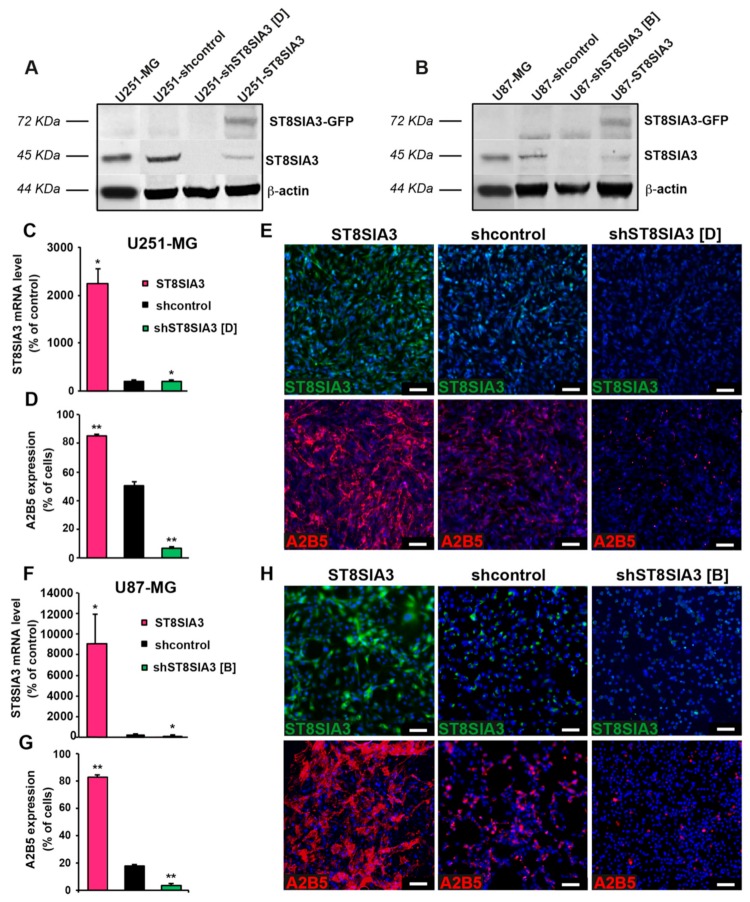
Expression of ST8 alpha-N-acetyl-neuraminidase α-2,8-sialyltransferase 3 (ST8SIA3) drives A2B5 immunoreactivity. (**A**) Western blot analysis of ST8SIA3-GFP (72 KDa) and endogenous ST8SIA3 (45 KDa) in U251-MG, U251-shcontrol, U251-shST8SIA3 [D], and U251-ST8SIA3 cell lines. The expression level of β-actin (44 KDa) was used as a loading control. Ratios of ST8SIA3/β-actin relative to U251-shcontrol from 3 independent experiments are presented under the blot. (**B**) Western blot analysis of ST8SIA3-GFP and endogenous ST8SIA3 in U87-MG, U87-shcontrol, U87-shST8SIA3 [B], and U87-ST8SIA3 cell lines. The expression level of β-actin was used as a loading control. Ratios of ST8SIA3/β-actin relative to U87-shcontrol from 3 independent experiments are presented under the blot. (**C**) RT-qPCR demonstration of ST8SIA3 overexpression in the U251-ST8SIA3 cell line after lentiviral infection and comparison with U251-shcontrol and U251-shST8SIA3 [D] cell lines. Data from five independent experiments are expressed as mean ± SEM. (**D**) Flow cytometry analysis of A2B5 in the U251-ST8SIA3 cell line and comparison with U251-shcontrol and U251-shST8SIA3 [D] cell lines. Data from six independent analyses are expressed as mean ± SEM. (**E**) Immunofluorescence staining of ST8SIA3 (green) and A2B5 (red) in U251-ST8SIA3, U251-shcontrol, and U251-shST8SIA3 [D] cell lines. Hoechst staining of the cell nuclei (blue) is also shown. Scale bar = 50 μm. (**F**) RT-qPCR demonstration of ST8SIA3 overexpression in the U87-ST8SIA3 cell line after lentiviral infection and comparison with U87-shcontrol and U87-shST8SIA3 [B] cell lines. (**G**) Flow cytometry analysis of A2B5 in the U87-ST8SIA3 cell line and comparison with U87-shcontrol and U87-shST8SIA3 [D] cell lines. (**H**) Immunofluorescence staining of ST8SIA3 (green) and A2B5 (red) in U87-ST8SIA3, U87-shcontrol, and U87-shST8SIA3 [B] cell lines. Cell nuclei are in blue. Scale bar = 50 μm. Non-parametric Wilcoxon-Mann-Whitney test shows * p < 0.05 and ** p < 0.01.

In order to verify whether ST8SIA3 expression drives the expression of antigens exhibiting A2B5 immunoreactivity, we first used GBM cell lines expressing mild (U251-MG, 50.25% ± 3.06%) and low (U87-MG, 17.5% ± 0.96%) levels of A2B5 immunoreactivity. The *ST8SIA3* gene was stably overexpressed by lentiviral infection or silenced by using shRNA technology in these two cell lines. Manipulated cell lines were analyzed by Western blot for ST8SIA3 and ST8SIA3-GFP expression (Figure 1A,B). ST8SIA3 mRNA was significantly increased in ST8SIA3-overexpressing cells (U251-ST8SIA3: 2239 ± 466 A.U.; U87-ST8SIA3: 9064 ± 2908 A.U., % of control RNA) when compared to shcontrol cell lines (U251-shcontrol: 51.12 ± 2.2 A.U., *p* < 0.05; U87-shcontrol: 0.2 ± 0.01 A.U., *p* < 0.05) and to the shST8SIA3 cells (U251-shST8SIA3: 11.12 ± 1.1, *p* < 0.05; U87-shST8SIA3: 0.07 ± 0.01, *p* < 0.05) (Figure 1C,F). At the protein level, ST8SIA3 was increased in the ST8SIA3-overexpressing cells and decreased in the shST8SIA3 cells (Figure 1E,H). A2B5 quantification by flow cytometry revealed a highly significant increase of A2B5 immunoreactivity in ST8SIA3-overexpressing cells as compared to the control cell line (U251-ST8SIA3: 85.13% ± 2.59%, *p* < 0.01; U87-ST8SIA3: 82.62% ± 1.86%, *p* < 0.01) and a drastic reduction of A2B5-positive cells in shST8SIA3 cells (U251-shST8SIA3: 2.7% ± 1.1%, *p* < 0.01; U87-shST8SIA3: 1.6% ± 0.2%, *p* < 0.01) (Figure 1D,G). By immunofluorescence, A2B5 was clearly highlighted when ST8SIA3 was overexpressed and abolished in U251-shST8SIA3 and U87-shST8SIA3 (Figure 1E,H). Therefore, ST8SIA3 drives A2B5 immunoreactivity in GBM cells.

### 2.2. Overexpression and Silencing of ST8SIA3 in Mild- and Low-A2B5-Expressing Cells Modulate Cell Death, Cell Proliferation, and Migration in GBM in Vitro

**Figure 2:**
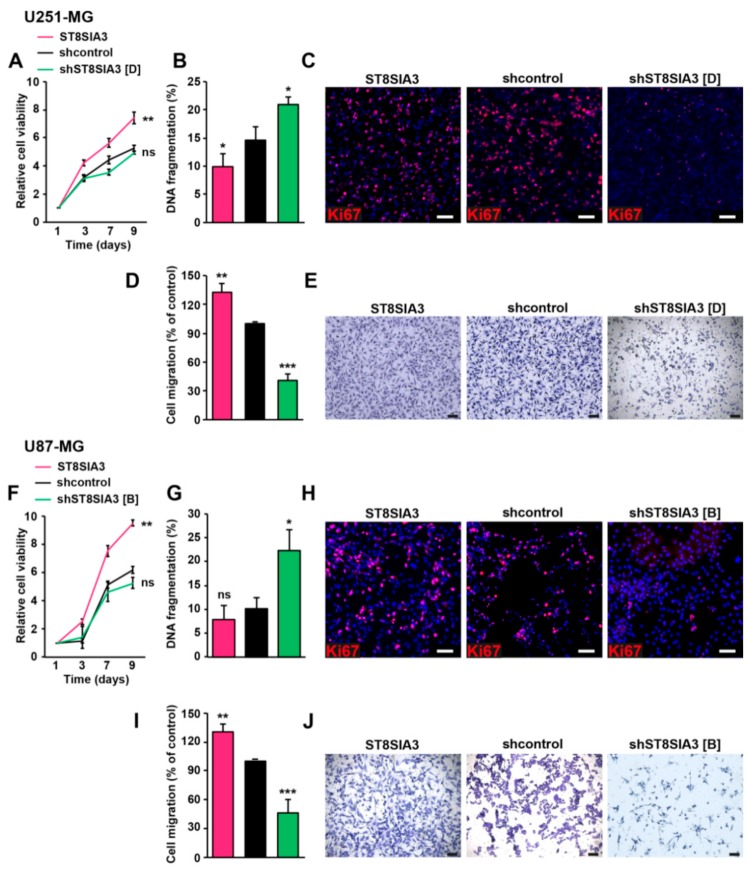
ST8SIA3 modulates in vitro cell death, proliferation, and migration. (**A**,**F**) Colorimetric MTT assay (3-(4,5-dimethylthiazol-2yl)-diphenyl tetrazolium bromide) was performed to compare cell viability (*n* = 5 for each cell line). (**B**,**G**) DNA fragmentation (sub/G_1_) was determined by flow cytometry of propidium-iodide-stained nuclei. Data are expressed as mean ± SEM deduced from five independent experiments. (**C**,**H**) Immunofluorescence staining of the proliferation marker Ki67 (red) in ST8SIA3, shcontrol, and shST8SIA3 cell lines. Cell nuclei are counterstained in blue. Scale bar = 50 μm. (**D**,**I**) Quantification of cell migration using the transwell assay determined by the optical density of SDS-solubilized crystal violet. The mean + SEM values of four independent experiments, each performed in duplicate, are shown. (**E**,**J**) Representative phase contrast images of migrating cells. Scale bar = 50 μm. Non-parametric Wilcoxon-Mann-Whitney test shows * *p* < 0.05, ** *p* < 0.01, and *** *p* < 0.001. ns: non-significant.

We first quantified the impact of ST8SIA3 expression on cell viability. ST8SIA3 overexpression significantly increased cell viability over time as compared to shcontrol cells (U251-ST8SIA3, *p* < 0.01; U87-ST8SIA3, *p* < 0.01). However, no variation was recorded between shST8SIA3 cells and shcontrol cells (Figure 2A,F). DNA fragmentation (sub/G_1_) analyses showed a decrease of cell death in ST8SIA3-overexpressing cells as compared to control cells and, to the contrary, a significant increase of cell death in shST8SIA3 cell lines (Figure 2B,G). Ki67 immunofluorescence showed that cells overexpressing ST8SIA3 proliferated more than the control cells, and inversely, the shST8SIA3 cells proliferated less than the control cells (Figure 2C,H). We then analyzed the effect of ST8SIA3 expression on cell migration. The overexpression of ST8SIA3 significantly boosted the migration ability of the cells as compared to shcontrol cells (U251-ST8SIA3, *p* < 0.01; U87-ST8SIA3, *p* < 0.01). Conversely, the silencing of ST8SIA3 abrogated migration ability (U251-shST8SIA3, *p* < 0.001; U87-shST8SIA3, *p* < 0.001; Figure 2D,E,I,J). Altogether our results show that A2B5 immunoreactivity is positively correlated with cell viability, proliferation, and migration in GBM cell lines.

### 2.3. ST8SIA3 Overexpression in Low-A2B5-Expressing Cells Increases Clonogenicity and Tumorigenicity

**Figure 3:**
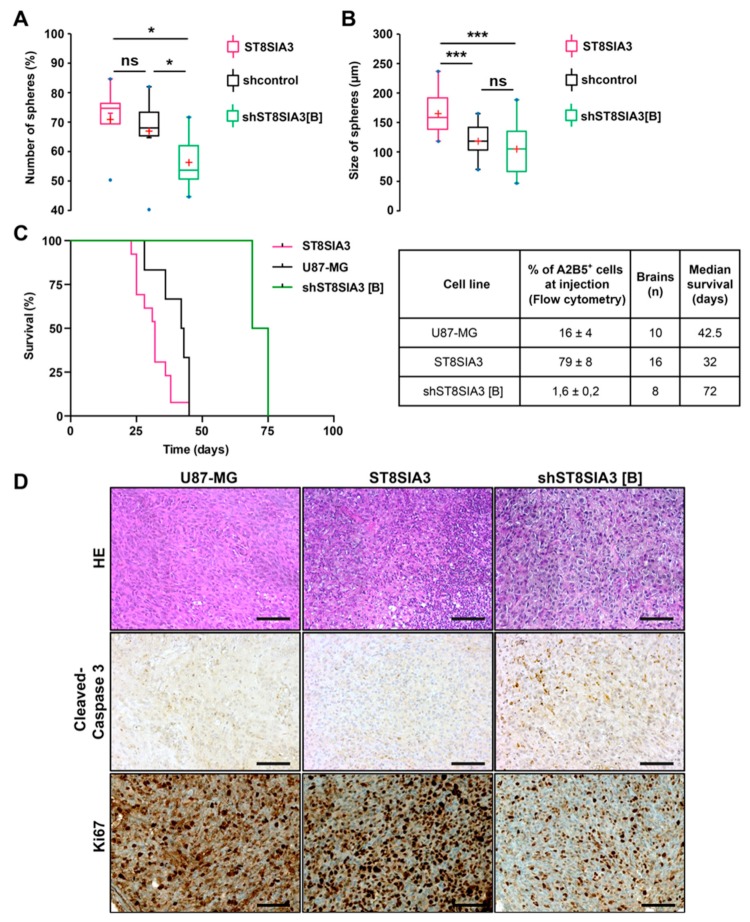
ST8SIA3 enhances in vitro clonogenicity and in vivo tumorigenicity. (**A**) Self-renewal capacity was evaluated by limiting dilution assay in 96-well plates. Box plots show the percentage of spheres formed after 8 days of culture for U87-shcontrol, U87-ST8SIA3, and U87-shST8SIA3 [B] cell lines. (**B**) Mean diameter of spheres recorded for U87-shcontrol, U87-ST8SIA3, and U87-shST8SIA3 [B] after 8 days of culture. Non-parametric Wilcoxon–Mann–Whitney test shows * *p* < 0.05 and *** *p* < 0.001. ns: non-significant. The lower and upper edges of each box represent the first and third quartiles, respectively, while the horizontal line within the box indicates the median. The vertical length of the box represents the interquartile range (IQR). The most extreme sample values (within a distance of 1.5 IQR from the median) are the endpoints of the extending lines. (**C**) Kaplan–Meier survival plot of mice intracerebrally grafted with U87-MG, U87-ST8SIA3, or U87-shST8SIA3 [B] cells. Time is expressed in days since time of cell graft. Groups were compared using the Log-rank test. The table recaps the percentage of A2B5^+^ cells before injection, number of brains analyzed, and median survival of mice. (**D**) Representative hematoxylin–eosin (HE), cleaved-caspase 3, and Ki67 staining of tumors obtained after U87-MG, U87-ST8SIA3, or U87-shST8SIA3 [B] orthotopic injections. Scale bar: 100 μm.

By limiting dilution assay in serum-free medium, the U87-ST8SIA3 cells exhibited higher clonogenicity than U87-shcontrol and U87-shST8SIA3 (76% versus 68% versus 52%) (Figure 3A). These differences were statistically significant between U87-ST8SIA3 and U87-shST8SIA3 (*p* = 0.018) and between U87-shcontrol and U87-shST8SIA3 (*p* = 0.041) but not between U87-ST8SIA3 and U87-shcontrol (*p* = 0.293). Moreover, the diameter of spheres was significantly larger for U87-ST8SIA3 (165.9 µm) than for U87-shcontrol (118.7 µm) and U87-shST8SIA3 (99.0 µm) (Figure 3B). These differences were statistically significant between U87-ST8SIA3 and U87-shST8SIA3 (*p* = 0.001) and between U87-ST8SIA3 and U87-shcontrol (*p* = 0.001) but not between U87-shcontrol and U87-shST8SIA3 (*p* = 0.334). These results indicate that cells overexpressing ST8SIA3 gain clonogenic properties and increase their self-renewal and proliferation rate.

Then, to determine whether the ST8SIA3 expression level could influence tumorigenicity, we performed stereotactic orthotopic intracranial injections of U87-MG, U87-ST8SIA3, and U87-shST8SIA3 cells. Expression of A2B5 was analyzed by flow cytometry immediately prior to injection. At the end of the experiment, extracted brains were macroscopically and microscopically analyzed for the presence of a tumor and its histological characterization. Interestingly, ST8SIA3 overexpression shortened the overall survival and decreased 1.3-fold the median survival from 42.5 days (range 36–45) for U87-MG to 32 days (range 23–45) for U87-ST8SIA3-tumor-bearing mice (*p* < 0.05) (Figure 3C). Conversely, a significant improvement of median survival was measured for U87-shST8SIA3-bearing mice of 72 days (range 69–75) compared to 42.5 days in U87-MG-ones (*p* < 0.05), and as expected, median survival was higher when compared to U87-ST8SIA3 mice (32 days, *p* = 0.02). It is worth noting that in the U87-shST8SIA3 group, only two out of eight mice developed a tumor. The data unambiguously demonstrate that in vivo, the A2B5 expression level negatively correlates with overall survival. Histologically, tumors did not differ between the three groups of mice (Figure 3D). They were dense and mostly circumscribed. However, a clear difference was revealed by cleaved caspase 3 immunohistochemistry. The U87-shST8SIA3 tumors showed significant higher staining as compared to U87-MG or U87-ST8SIA3 tumors (Figure 3D). In addition, U87-shST8SIA3 tumors demonstrated a lower Ki67 proliferation index (30% to 40%) in comparison to U87-MG (around 80%) or U87-ST8SIA3 (more than 90%) tumors (Figure 3D).

### 2.4. Suppression of A2B5 Immunoreactivity in High-A2b5-Expressing Cells Induces a Decrease in Cell Proliferation, Migration, and Clonogenicity

As high-A2B5-expressing cells, we used GBM CSCs developed in our laboratory [21]. To determine whether A2B5 removal could reverse the stem-like cell phenotype, we silenced ST8SIA3 in four GBM CSCs: GBM6, GBM9, GBM27, and GBM40. After several infection trials with the shST8SIA3 in these four cell lines, we succeeded in producing GBM9-shST8SIA3 spheres with undetectable A2B5 immunoreactivity as compared to native GBM9 and GBM9-shcontrol cells (Supplementary Figure S1A). However, GBM9-shST8SIA3 culture could not be maintained over time, and self-renewal stopped after three passages. Assays performed on these GBM9-shST8SIA3 cells revealed their drastically impaired migration ability compared to GBM9-shcontrol (Supplementary Figure S1B). In GBM6, 27, and 40, ST8SIA3 silencing invariably led to cell death, strongly suggesting that A2B5 is necessary to maintain cell growth. 

**Figure 4:**
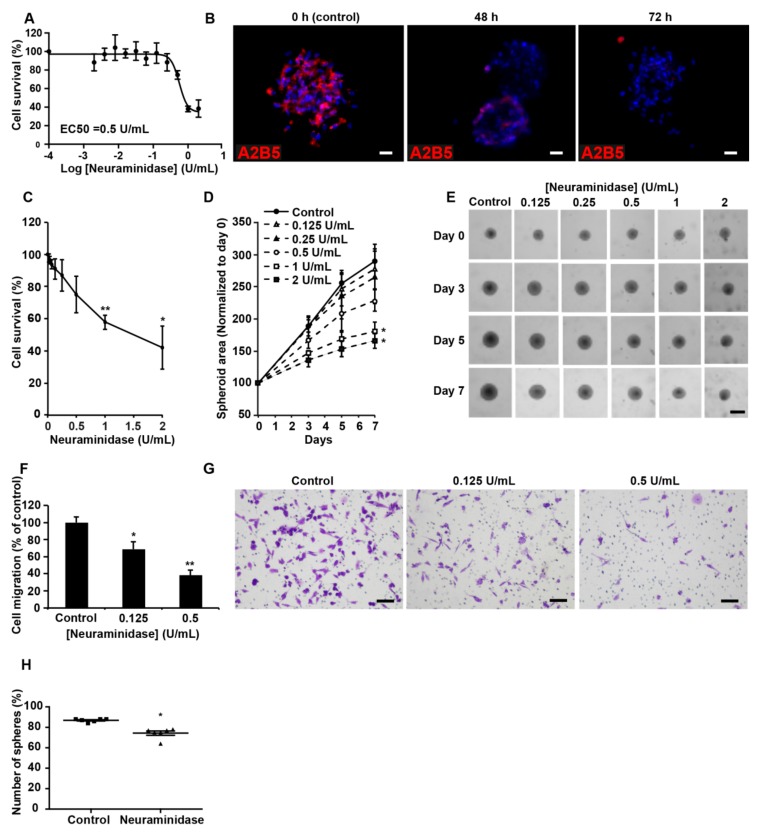
Suppression of A2B5 immunoreactivity in high-A2B5-expressing cells induces a decrease in cell proliferation, migration, and clonogenicity. (**A**) A dose–response cytotoxicity assay of neuraminidase (10^−4^ to 1 U/mL) was conducted for 72 h to assess the viability of GBM9 cells grown as a 2D monolayer (*n* = 3). (**B**) A2B5 staining (in red) on the GBM9 sphere control or that treated with 0.5 U/mL neuraminidase (EC50) for 48 and 72 h. (**C**) A dose-response cytotoxicity assay of neuraminidase (0 to 2 U/mL) was conducted for 7 days to assess the viability of GBM9 cells grown as 3D spheroids (*n* = 3). (**D**) The GBM9 spheroid area was measured during the 7 days after neuraminidase treatment and normalized to Day 0. (**E**) Representative phase contrast images at Days 0, 3, 5, and 7 of the control and treated GBM9 spheroids. (**F**) Quantification of GBM9 cell migration using the transwell assay after neuraminidase treatment at 0.125 and 0.5 U/mL (EC50) for 6 h. The mean + SEM values of four independent experiments, each performed in duplicate, are shown. (**G**) Representative phase contrast images of control and treated migrating GBM9 cells. Scale bar = 50 μm. (**H**) Self-renewal capacity was evaluated by limiting dilution assay in 96-well plates. The scatter plot shows the percentage of spheres formed after 8 days of culture of GBM9 cells treated or not with 0.5 U/mL neuraminidase (EC50). Non-parametric Wilcoxon-Mann-Whitney test shows * *p* < 0.05, ** *p* < 0.01.

As an alternative to ST8SIA3 silencing, we decreased A2B5 expression at the surface of the high- A2B5-expressing cells by using the hydrolase neuraminidase, which removes α-2,8 bonds [9]. A dose–response cytotoxicity assay of neuraminidase (10^−4^ to 1 U/mL) was conducted for 72 h on GBM6, GBM9, GBM27, and GBM40 CSC lines (Figure 4 and Supplementary Figure S2). The enzyme concentrations to reduce viability by 50% (EC50) were determined for each cell line. The EC50 values were 0.56 U/mL for GBM9 (Figure 4A), 0.98 U/mL for GBM6, 0.47 U/mL for GBM27, and 0.96 U/mL for GBM40 (Supplementary Figure S2A–C). Neuraminidase strongly decreased A2B5 expression in GBM9 from 48 h to 72 h of treatment (Figure 4B). In GBM9 cells cultured as 3D spheroids, concentrations of neuraminidase at 1 and 2 U/mL deeply decreased cell viability (Figure 4C) and significantly reduced (by 50%) spheroid size as compared with the control (Figure 4D,E). The same dose effects and time effects on spheroid viability and volumes were obtained using spheroids produced from GBM6, GBM27, and GBM40 cells (Supplementary Figure S2D–I).

Finally, inhibition of cell migration by neuraminidase was assessed in GBM CSCs. In GBM9, a statistically significant dose effect was detected even at low concentration (0.125 U/mL) (Figure 4F,G). GBM6, GBM27, and GBM40 cell migration was also strongly reduced in presence of neuraminidase (Supplementary Figure S2J–L). In a 7-day subsphere-forming assay, neuraminidase at the EC50 concentration significantly impaired the formation of spheres (74%) when compared with a non-treated GBM9 cell line (86%, *p* < 0.05, Figure 4H), the high clonogenicity ability of which has been previously described [21]. Similar observations were made for GBM6, GBM27, and GBM40 (Supplementary Figure S2M–O). U251-MG cells were used to compare shST8SIA3 and neuraminidase effects (Supplementary Figure S3).

### 2.5. Neuraminidase Effects in Human GBM Explants

**Figure 5:**
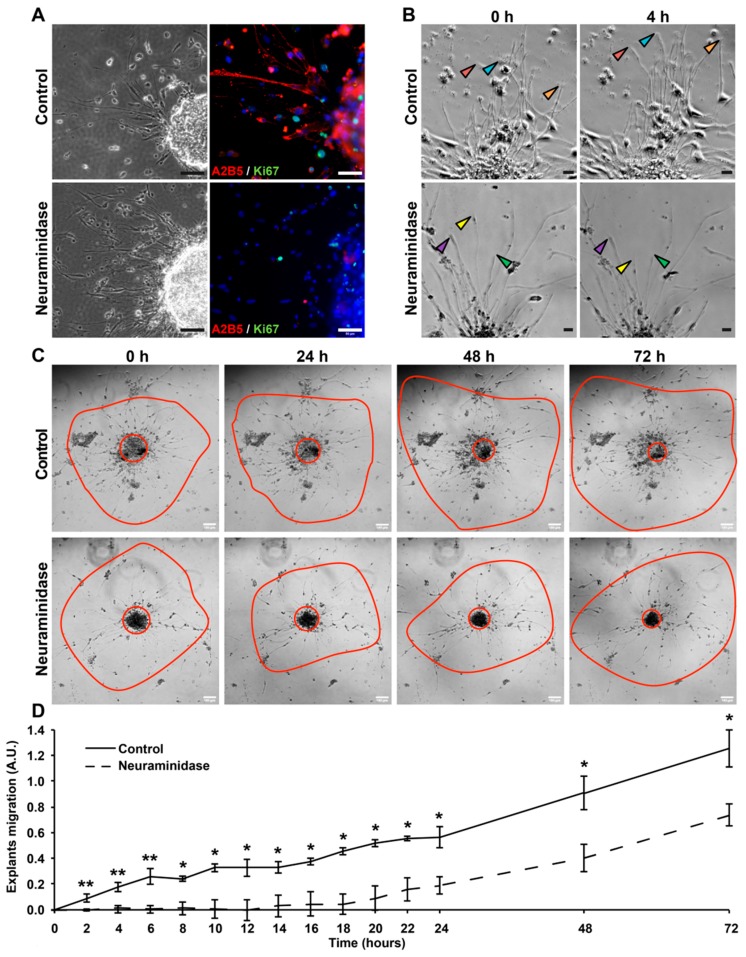
Neuraminidase affects the migration of human GBM explants. (**A**) Phase-contrast images and immunofluorescent double-staining of A2B5 (in red) and Ki67 (in green) of control and 72 h treated (1 U/mL neuraminidase) explants. Cell nuclei are counterstained in blue. Scale bar = 50 μm. (**B**) Using time-lapse video microscopy at regular intervals, cells migrating radially from explants in basic conditions or with 1 U/mL neuraminidase were accurately observed for 72 h. Arrows allow us to follow the migration of some cells. Scale bar = 50 μm. (**C**) Representative phase contrast images taken over time of control and treated (1 U/mL neuraminidase) explants. For each explant, the central red circle allows us to visualize the explant’s core, while the outer red line shows the cell migration area. Scale bar = 100 μm. (**D**) The distance of migrating cells from the explant’s core was quantified in basic conditions or with 1 U/mL neuraminidase (*n* = 3 experiments). Non-parametric Wilcoxon–Mann–Whitney test shows * *p* < 0.05 and ** *p* < 0.01. ns: non-significant.

To determine whether fresh human GBM tissue could respond to neuraminidase as shown previously in GBM cell models, we performed explant cultures. First, we analyzed the effect of neuraminidase at 1 U/mL for 72 h. As shown in Figure 5A, after 72 h of treatment, A2B5 expression was lost in neuraminidase-treated explants. A2B5 removal decreased proliferation as shown by the Ki67 expression. By using time-lapse video microscopy, we tracked cell migration in the explants (Supplementary Figure S5, Videos 1,2). We followed the behavior of cells radially migrating from the core. At early stages of neuraminidase treatment, cellular extensions of migrating cells were retracting, whereas in control conditions, extensions stayed intact and continue to grow (see arrows, Figure 5B). We recorded the distances of migration of cells from the explant core during those 72 h, and neuraminidase treatment significantly reduced migration (Figure 5C,D). Altogether these results confirmed in human GBM tissues that neuraminidase treatment leads to a decrease in cell proliferation and migration. 

## 3. Discussion

Our interest in GBM, gliomagenesis, and CSCs brought us to focus on sialogangliosides recognized by the monoclonal antibody A2B5. Experimental evidence strongly suggests that A2B5-positive cells isolated from GBM represent GBM initiating cells [8,14,15,21]. An open question was whether these cell surface sialogangliosides, beside their interest as markers, play an active role in influencing GBM cell properties. To answer this question, we used two strategies: overexpressing versus suppressing the synthesis of the A2B5 epitope by manipulating the key enzyme ST8SIA3 and neuraminidase treatment which abrogates the cell surface A2B5 epitope. 

Introducing the ST8SIA3 coding sequence into low- and mild-level A2B5-expressing GBM cell lines resulted in a huge increase of A2B5 immunoreactivity and showed that the A2B5 immunoreactivity level was positively correlated with ST8SIA3 expression. Consequently, deep changes in cell behavior were recorded for proliferation and migration abilities. Moreover, we showed that U87-ST8SIA3 cells acquired clonogenic potential and were more tumorigenic in vivo. In contrast, when ST8SIA3 silencing was performed in these cells, they lost their ability to migrate in vitro and were less tumorigenic in vivo. A strong indication of a functional role of A2B5-bearing gangliosides in CSC came from the observation that ST8SIA3 silencing in high-A2B5-expressing CSC lines GBM6, GBM9, GBM27, and GBM40 prevented their maintenance in culture, likely by impairing cell growth and proliferation. To confirm this hypothesis, we reduced the A2B5 cell surface level in these GBM CSCs by using neuraminidase, a hydrolase which cleaves alpha 2–8 neuraminic acid bonds. The removal of the A2B5 epitope by neuraminidase in CSCs decreased survival, migration, and clonogenicity. It is worth noting that on U251-MG cells, similar effects were recorded with neuraminidase or shST8SIA3, confirming that these two approaches lead to a decrease in A2B5 immunoreactivity. Moreover, we also demonstrated that in an ex vivo human GBM model, A2B5 removal by neuraminidase blocked cell migration at early time points. Taken together, our results support a major role of the A2B5 epitope in cellular growth, proliferation, and migration. 

Furthermore, we showed that A2B5 is involved in tumor initiation. By transplanting U87-ST8SIA3 and U87-shST8SIA3, we gained insight about the role of A2B5 in tumor initiation and growth. U87-ST8SIA3 cells developed tumors faster than U87-MG. Therefore, by increasing A2B5 expression, tumor grafting was more efficient. Besides this, only two out of eight U87-shST8SIA3-injected mice developed a tumor, and in these mice, tumor development was postponed, probably due to residual A2B5-positive cells in the grafted cell suspension. These results are in line with those observed with the GD3 ganglioside, which is the main precursor of GT3 that carries the A2B5 epitope. Introduction of the cDNA of GD3 synthase (also called ST8SIA1) into the U251-MG cell line induces high levels of GD3 and GD2 on the cell surface, leading to an increase of invasion and mobility [7]. Moreover, Yeh et al. showed that GD3 and GD3 synthase are key drivers of GBM stem cells and tumorigenicity [6]. Importantly, GD3 is also required for neurogenesis and long-term maintenance of neural stem cells in the postnatal mouse brain [22]. However, in these studies, the authors did not search for A2B5 expression. These observations could therefore be associated either to GD3 or to A2B5 expression.

The mechanisms by which the A2B5 epitope could act on cell proliferation, migration, clonogenicity, and tumorigenicity are worth exploring. Ganglioside head groups, protruding toward the extracellular space, significantly contribute to the cell glycocalyx and are major determinants of the features of the cell surface. In particular, they modulate complex interactions with other molecules sitting on the same cellular membrane (*cis* interactions) or soluble molecules present in the extracellular environment or with molecules associated with the surface of other cells (*trans* interactions) (reviewed in [2,23]). It is thus conceivable that the effects of A2B5 gangliosides are exerted by modulating the activity of receptors and their following signaling cascades [24].

Besides this, the A2B5 epitope, like other gangliosides, represents an attractive therapeutic target. GD2- and GD3-targeting immunotherapies are currently under clinical or preclinical investigations in several solid tumors [25] (reviewed in [24]). Several approaches have been proposed to target gangliosides, including cancer vaccination, monoclonal antibodies, and CAR-T (Chimeric Antigen Receptor T) cells. In GBM cells it has been reported that adjuvant monoclonal antibody 8B6, which targets O-acetyl GD2, impairs temozolomide resistance driven by glioma CSCs [5]. Of particular interest, the potent anti-tumor efficacy of anti-GD2 CAR T cells has recently been reported in mouse models of H3K27M+ midline gliomas, a highly infiltrative and fatal glioma [26]. In addition of immunotherapy targeting cell surface gangliosides, another attractive therapeutic approach, relies on the potential use of intracellular antibodies to prevent the translocation of the sialyltransferase from the endoplasmic reticulum to the Golgi apparatus. This approach has been successfully used in experimental models targeting ST8SiaII and ST8SiaIV in order to inhibit polysialylation of NCAM (Neural Cell Adhesion Molecule) in rhabdomyosarcoma tumor cells [27]. Furthermore, it is known that sialic acids are responsible for an immune-suppressive environment. It has been shown that the inhibition of sialylation through sialic acid mimetic drugs induced CD8+ T-cell-mediated killing of tumor cells in various murine tumor models [28]. The strong expression of highly sialylated gangliosides such as those recognized by the A2B5 antibody might explain to some extent the failure of clinical trials targeting immune checkpoint inhibitors in GBM (reviewed in [29]).

Altogether it is likely that targeting A2B5 in GBM cell lines is a promising therapeutic approach. Such a treatment might not only act on tumor cells but might also lead to a more permissive immune environment. This will pave the way for efficient treatments that combine sialic acid targeting and immune checkpoint inhibitors in GBM.

## 4. Materials and Methods 

### 4.1. Cell Lines and Reagents

U251-MG and U87-MG cell lines (American Type Culture Collection, Manassas, VA, USA) were cultured as an adherent monolayer in Dulbecco’s modified Eagle’s medium (DMEM, Life Technologies, Courtaboeuf, France) supplemented with 10% heat-inactivated fetal calf serum (FCS, Life Technologies), 50 U/mL penicillin, and 50 µg/mL streptomycin (Life Technologies) at 37 °C in a humidified atmosphere of 5% CO_2_ and 95% air. Confirmation of U251-MG and U87-MG identity was performed by LGC standards (Teddington, Middlesex, UK). GBM6, GBM9, GBM27, and GBM40 CSC lines, established at our laboratory on the basis of A2B5 expression from different human GBM tumor samples, exhibited features reminiscent of the clinical characteristics of the original tumors. They were maintained in culture as floating spheres in serum-free medium supplemented with EGF (Epidermal Growth Factor) and bFGF (basic Fibroblast Growth Factor) as previously described [14,21]. In these cell lines, the A2B5 level recorded by flow cytometry was usually 80%–90% [14]. All cells were tested monthly for the absence of mycoplasma. Neuraminidase (sialidase) from *Clostridium perfringens* was purchased from Sigma-Aldrich. It was dissolved at 50 U/mL in sterile water and stored at 4 °C until use. For neuraminidase treatment experiments, cells were grown either as monolayers (for CSC, on 10 µg/mL poly-DL-ornithine (Sigma-Aldrich, coated plates, Saint-Quentin Fallavier, France) or as spheres. Cells and spheres were treated at their EC50 (U251-MG and U87-MG: 0.091 U/mL; GBM6: 0.98 U/mL; GBM9: 0.56 U/mL; GBM27: 0.47 U/mL and GBM40: 0.96 U/mL; Figure 4A and Supplementary Figure S2A–C).

### 4.2. Manipulation of ST8SIA3 Expression

#### 4.2.1. Overexpression of ST8SIA3

The coding region of ST8SIA3 (Unigene ID: Hs.23172) was subcloned into the lentiviral vector pRRLSIN.cPPT.PGK-GFP.WPRE (referred to as pRRL, kindly provided by Trono, University of Geneva, Geneva, Switzerland). This was used for the generation of stable ST8SIA3-expressing U251-MG and U87-MG cell lines using the conventional protocol. Lentiviral particle preparation and infection of the human cells with viral particles were performed according to Mathieu et al. [30]. The expression level of ST8SIA3 was monitored by RT-qPCR and/or immunofluorescence.

#### 4.2.2. Down-Regulation of ST8SIA3: SHST8SIA3

In our experiments, the ST8SIA3 shRNA expression cassette consisted of a 29 bp target-gene-specific sequence (cacacttcagcaactgtgaccaggacatt (A) or gctgctggccgctcaatggaccgatttcc (B) or aacggctgagcacaggtattcttatgtac (C) or ctctgtcacactgtgcctaagaactccaa (D)), a 7 bp loop, and another 29 bp reverse complementary sequence, all under human U6 promoter in a pGFP-C-shLenti vector also expressing enhanced green fluorescent protein (GFP) under CMV (cytomegalovirus) promoter (OriGene Technologies, Rockville, MD, USA). The preparation of shST8SIA3 lentiviral particules was performed according to instructions from OriGene Technologies. In brief, HEK293T cells were co-transfected with pGFP-C-shLenti together with the packaging plasmid and MEGA-TRAN. Lentivirus-containing supernatants were harvested, filtered through 0.22 μm cellulose acetate filters (Merck KGaA, Darmstadt, Germany), aliquoted, and stored at −80 °C until use. For lentiviral infection, the culture medium was removed when U251-MG and U87-MG cells were at 80% confluence. They were washed with PBS and treated with the virus-containing medium combined with 5 μg/mL polybrene (Sigma-Aldrich). After 72 h of transduction, 0.2 µg/mL puromycin (Life Technologies) was added to the medium for stable cell line selection. The lentiviral vector carrying a 29 mer scrambled sequence cassette was used as a negative control (OriGene Technologies) (Supplementary Figure S4). For shST8SIA3-GBM6, -GBM9, -GBM27, and -GBM40 production, cells were seeded beforehand on 10 µg/mL poly-DL-ornithin with serum-free medium. Under these conditions, they were able to adhere to the plastic without differentiating [14]. At 80% confluence, infection was performed as explained above.

### 4.3. Immunostainings

U251-MG and U87-MG cell lines were grown as a monolayer on glass coverslips in 24-well plates in DMEM with 10% FCS. For GBM CSCs, immunofluorescence experiments were performed on 8-day-old spheres or on cells cultured on poly-DL-ornithin as explained above. Primary antibodies A2B5 (mouse IgM, clone 105, 1/1000, kindly provided by G. Rougon, Marseille, France), anti-ST8SIA3 (rabbit IgG, 1/1000, Sigma-Aldrich), and anti-Ki67 (mouse IgG, clone Mib1, Dako, Les Ulis, France) were incubated for 1 h at room temperature. Fluorochrome-conjugated secondary antibodies Alexa fluor 568 anti-mouse IgM, Alexa fluor 488 anti-rabbit IgG, Alexa fluor 568 anti-mouse IgG, or Alexa fluor 568 anti-goat IgG (Molecular Probes, Eugene, OR, USA) were incubated at 2 µg/mL for 1 h at room temperature together with Hoechst 33342. All the images were obtained using a Zeiss AXIO-Observer Z1 microscope (Carl Zeiss SAS, Marly-le-Roi, France). Conventional immunohistochemistry for cleaved caspase-3 (C92-605 clone, 1/500, BD Biosciences, Franklin Lake, NJ, USA ) and Ki67 (30-9 clone, Ventana Medical Systems, Illkirch, France) was performed using the Benchmark XT automate (Ventana Medical Systems) according to the manufacturer’s instructions. Sections without primary antibody served as a negative control.

### 4.4. Flow Cytometry

Flow cytometry was performed using FACS Calibur (BD Biosciences) on dissociated cells to quantify the number of cells expressing A2B5 (APC (Allophycocyanin)-labelled IgM, clone 105, Miltenyi Biotec, Bergisch Gladbach, Germany) using a previously described standard procedure [14,21]. Data were analyzed using FlowJo software (Tree Star, Inc., Ashland, OR, USA).

### 4.5. Protein Extraction and Western Blotting 

Experiments were performed as previously described [31] using the following primary antibodies: anti-ST8SIA3 (rabbit IgG, 1/1000, Sigma-Aldrich) or anti-β-actin (mouse IgG, 1/5000, clone AC-15, Merck KGaA) as an internal loading control. 

### 4.6. Viability/Cytotoxicity Assay

For two-dimensional (2D) experiments, cell viability was measured using the colorimetric MTT assay (3-(4,5-dimethylthiazol-2yl)-diphenyl tetrazolium bromide, Sigma-Aldrich) as previously described [32]. For 3D experiments, cell viability was assessed by resazurin reduction (alamarblue, Thermo Fisher Scientific, Vellebon-sur-Yvette, France) after 7 days of treatment as previously described [33]. 

### 4.7. DNA Fragmentation

Fluorescence-activated cell sorting (FACSCalibur) analysis of the DNA fragmentation of propidium-iodide-stained nuclei was performed as described in [34].

### 4.8. In Vitro Migration Assay 

A total of 50,000 cells was seeded in serum-free DMEM on the filter membranes of 8 μm transwell migration chambers (Corning Inc., Amsterdam, The Netherlands). DMEM with 10% FCS was added to the wells under the filter and cells were allowed to migrate for 6 h. Transmigrated cells were fixed in 4% paraformaldehyde and stained with 0.4% crystal violet, and membranes were photographed (Carl Zeiss SAS™). For quantification, cells were counted or crystal violet was solubilized in SDS 1% and absorbance was measured at 550 nm using an Elx800 microplate reader (Bio-Tek, Colmar, France).

### 4.9. Limiting Dilution Assay 

The U87-MG cell line was plated in limiting dilution conditions in 96-well plates. Clonal frequency was quantified at Day 8. At the same time, the diameter of spheres was recorded as previously described [14,21]. For GBM CSCs, the number of spheres was reported relative to the total number of cells seeded to evaluate the sphere formation property after 8 days of neuraminidase treatment.

### 4.10. Reverse Transcription, Real-Time Quantitative PCR Analysis (RT-qPCR)

Total RNA extraction and gene expression analysis were performed as previously described [32]. Ribosomal 18S and glyceraldehyde-3-phosphate-deshydrogenase (GAPDH) were used as reference genes. Forward and reverse primers for each gene are listed below: 18S: 5′- CTACCACATCCAAGGAAGGCA-3′, 5′-TTTTTCGTCAACTACCTCCCCG-3′; GAPDH: 5′-CAAATTCCATGGCACCGTC-3′, 5′-CCCACTTGATTTTGGAGGGA-3′; and ST8SIA3: 5′-TCCCTGCATTTTTCTTCCAC-3′, 5′-ACGGCCAAAATCCATACAAGC-3′. The PCR conditions were 5 min at 95 °C followed by 45 cycles of 15 s at 95 °C and 30 s at 65 °C. The relative expression of the target gene transcript was calculated as previously described [35] using human brain total RNA as the control (Agilent Technologies™, Les Ulis, France). Measurements were performed in triplicate. Results are expressed in arbitrary units (A.U.) as a percentage of control RNA.

### 4.11. Intracranial Injections 

All experimental procedures and animal care were carried out in accordance with the guidelines of the French Government, reviewed and approved by the Regional Institutional Committee for Ethics on Animal Experiments under the authorization number 04633.01. Six-week-old athymic female nude mice (Envigo) were anesthetized, and 50,000 cells of U87-MG (*n* = 18), U87-ST8SIA3 (*n* = 18), and U87-shST8SIA3 [B] (*n* = 10) were stereotactically injected into the corpus callosum (1 mm anterior to bregma, −1 mm lateral, and −2 mm deep in the cortex surface) as previously described [14]. The body weight and clinical status of mice were recorded every 2 days. Mice were euthanatized when they exhibited more than a 20% reduction from their initial body weight or significant neurological or motor deficit. Brains were immediately extracted, fixed in formalin, paraffin embedded, sectioned at 4 µm thickness, and processed to validate tumor development and for standard histology (hematoxylin-eosin staining).

### 4.12. Explant Cultures of Human GBM Tissue

Primary human GBM samples (*n* = 5) were collected at Assistance Publique-Hôpitaux de Marseille (AP-HM) after surgery and placed in Hank’s Balanced Salt Solution. Tissues were mechanically cut into 300 µm pieces and plated in 24-well plates (BD Biosciences) on coverslips precoated with poly-L-lysine (10 mg/mL; Sigma-Aldrich). Serum-free medium was supplemented with 0.4% methylcellulose (Sigma-Aldrich). Explant cultures were then incubated at 37 °C in a humidified atmosphere of 5% CO_2_ and 95% air. After 7 days of culture, explants were treated with 1 U/mL neuraminidase. Immunofluorescence was performed 72 h later.

Time-lapse video microscopy was used to analyze explant migration. After the 7 days of culture, explants were treated or not with 1 u/mL of neuraminidase and were transferred to a Zeiss AXIO-Observer Z1 microscope (Carl Zeiss SAS) equipped with a CO_2_ chamber and maintained at 37 °C. Images of the explants were captured every 10 min for periods of 72 h using a 5× objective in order to obtain a quantitative analysis of the cell migration. Quantifications of the area core and the total area of explants were performed using ImageJ software (National Institutes of Health). Quantifications are represented by the (total area)/(core area) ratio and data were standardized using the H0 ratio. Data from three distinct GBM samples are presented and expressed as mean ± SEM.

### 4.13. Statistical Analyses

The non-parametric Wilcoxon-Mann-Whitney test was conducted using XLSTAT 2013 software (Addinsoft, Paris, France) to analyze cell migration and proliferation rates, flow cytometry, DNA fragmentation, and clonogenicity. Overall survival curves of intracranial xenografted mice were obtained according to the Kaplan-Meier method and compared using the Log-rank test using GraphPad Prism4 software (GraphPad, Inc., San Diego, CA, USA). All statistical tests were two-sided, and the threshold for statistical significance was *p* < 0.05. Significances are denoted as follows: *** *p* < 0.001, ** *p* < 0.01, * *p* < 0.05. 

## 5. Conclusions

In conclusion, our findings prove the crucial role of the A2B5 epitope in the promotion of proliferation, migration, clonogenicity, and tumorigenesis, indicating that it is an attractive therapeutic target for glioblastomas.

## Supplementary Materials

The following are available online at https://www.mdpi.com/2072-6694/11/9/1267/s1, Figure S1: ST8SIA3 silencing in high-A2B5-expressing cells leads to cell death. Figure S2: Suppression of A2B5 immunoreactivity in GBM6, GBM27, and GBM40 by neuraminidase impairs cell proliferation, migration, and clonogenicity. Figure S3: Suppression of A2B5 immunoreactivity in U251-MG cells by neuraminidase induces decrease in cell proliferation, migration, and clonogenicity. Figure S4: Western blot analysis of ST8SIA3 in shST8SIA3 cell clones. Figure S5: Time-lapse of the migration of GBM explants over 24 h, untreated (Video 1) or treated with neuraminidase (1 U/mL) (Video 2).

## References

[1] Louis D.N., Perry A., Reifenberger G., von Deimling A., Figarella-Branger D., Cavenee W.K., Ohgaki H., Wiestler O.D., Kleihues P., Ellison D.W. (2016). The 2016 World Health Organization Classification of Tumors of the Central Nervous System: A summary. Acta Neuropathol..

[2] Schnaar R.L., Gerardy-Schahn R., Hildebrandt H. (2014). Sialic acids in the brain: Gangliosides and polysialic acid in nervous system development, stability, disease, and regeneration. Physiol. Rev..

[3] Wagener R., Rohn G., Schillinger G., Schroder R., Kobbe B., Ernestus R.I. (1999). Ganglioside profiles in human gliomas: Quantification by microbore high performance liquid chromatography and correlation to histomorphology and grading. Acta Neurochir. (Wien).

[4] Merzak A., Koochekpour S., Pilkington G.J. (1994). Cell surface gangliosides are involved in the control of human glioma cell invasion in vitro. Neurosci. Lett..

[5] Fleurence J., Cochonneau D., Fougeray S., Oliver L., Geraldo F., Terme M., Dorvillius M., Loussouarn D., Vallette F., Paris F. (2016). Targeting and killing glioblastoma with monoclonal antibody to O-acetyl GD2 ganglioside. Oncotarget.

[6] Yeh S.C., Wang P.Y., Lou Y.W., Khoo K.H., Hsiao M., Hsu T.L., Wong C.H. (2016). Glycolipid GD3 and GD3 synthase are key drivers for glioblastoma stem cells and tumorigenicity. Proc. Natl. Acad. Sci. USA.

[7] Iwasawa T., Zhang P., Ohkawa Y., Momota H., Wakabayashi T., Ohmi Y., Bhuiyan R.H., Furukawa K., Furukawa K. (2018). Enhancement of malignant properties of human glioma cells by ganglioside GD3/GD2. Int. J. Oncol..

[8] Colin C., Baeza N., Tong S., Bouvier C., Quilichini B., Durbec P., Figarella-Branger D. (2006). In vitro identification and functional characterization of glial precursor cells in human gliomas. Neuropathol. Appl. Neurobiol..

[9] Eisenbarth G.S., Walsh F.S., Nirenberg M. (1979). Monoclonal antibody to a plasma membrane antigen of neurons. Proc. Natl. Acad. Sci. USA.

[10] Saito M., Kitamura H., Sugiyama K. (2001). The specificity of monoclonal antibody A2B5 to c-series gangliosides. J. Neurochem..

[11] Schnaar R.L. (2016). Gangliosides of the Vertebrate Nervous System. J. Mol. Biol..

[12] Nunes M.C., Roy N.S., Keyoung H.M., Goodman R.R., McKhann G., Jiang L., Kang J., Nedergaard M., Goldman S.A. (2003). Identification and isolation of multipotential neural progenitor cells from the subcortical white matter of the adult human brain. Nat. Med..

[13] Yanagisawa M. (2011). Stem cell glycolipids. Neurochem. Res..

[14] Tchoghandjian A., Baeza N., Colin C., Cayre M., Metellus P., Beclin C., Ouafik L., Figarella-Branger D. (2010). A2B5 cells from human glioblastoma have cancer stem cell properties. Brain Pathol..

[15] Ogden A.T., Waziri A.E., Lochhead R.A., Fusco D., Lopez K., Ellis J.A., Kang J., Assanah M., McKhann G.M., Sisti M.B. (2008). Identification of A2B5+CD133—Tumor-initiating cells in adult human gliomas. Neurosurgery.

[16] Sun T., Chen G., Li Y., Xie X., Zhou Y., Du Z. (2015). Aggressive invasion is observed in CD133(−)/A2B5(+) glioma-initiating cells. Oncol. Lett..

[17] Hakomori S. (2002). Glycosylation defining cancer malignancy: New wine in an old bottle. Proc. Natl. Acad. Sci. USA.

[18] Cazet A., Lefebvre J., Adriaenssens E., Julien S., Bobowski M., Grigoriadis A., Tutt A., Tulasne D., Le Bourhis X., Delannoy P. (2010). GD(3) synthase expression enhances proliferation and tumor growth of MDA-MB-231 breast cancer cells through c-Met activation. Mol. Cancer Res..

[19] Varki A., Gagneux P. (2012). Multifarious roles of sialic acids in immunity. Ann. N. Y. Acad. Sci..

[20] Xiao H., Woods E.C., Vukojicic P., Bertozzi C.R. (2016). Precision glycocalyx editing as a strategy for cancer immunotherapy. Proc. Natl. Acad. Sci. USA.

[21] Tchoghandjian A., Baeza-Kallee N., Beclin C., Metellus P., Colin C., Ducray F., Adelaide J., Rougon G., Figarella-Branger D. (2012). Cortical and subventricular zone glioblastoma-derived stem-like cells display different molecular profiles and differential in vitro and in vivo properties. Ann. Surg. Oncol..

[22] Wang J., Cheng A., Wakade C., Yu R.K. (2014). Ganglioside GD3 is required for neurogenesis and long-term maintenance of neural stem cells in the postnatal mouse brain. J. Neurosci..

[23] Ilic K., Auer B., Mlinac-Jerkovic K., Herrera-Molina R. (2019). Neuronal Signaling by Thy-1 in Nanodomains With Specific Ganglioside Composition: Shall We Open the Door to a New Complexity?. Front. Cell Dev. Biol..

[24] Mereiter S., Balmana M., Campos D., Gomes J., Reis C.A. (2019). Glycosylation in the Era of Cancer-Targeted Therapy: Where Are We Heading?. Cancer Cell.

[25] Ladenstein R., Potschger U., Valteau-Couanet D., Luksch R., Castel V., Yaniv I., Laureys G., Brock P., Michon J.M., Owens C. (2018). Interleukin 2 with anti-GD2 antibody ch14.18/CHO (dinutuximab beta) in patients with high-risk neuroblastoma (HR-NBL1/SIOPEN): A multicentre, randomised, phase 3 trial. Lancet Oncol..

[26] Mount C.W., Majzner R.G., Sundaresh S., Arnold E.P., Kadapakkam M., Haile S., Labanieh L., Hulleman E., Woo P.J., Rietberg S.P. (2018). Potent antitumor efficacy of anti-GD2 CAR T cells in H3-K27M(+) diffuse midline gliomas. Nat. Med..

[27] Somplatzki S., Muhlenhoff M., Kroger A., Gerardy-Schahn R., Boldicke T. (2017). Intrabodies against the Polysialyltransferases ST8SiaII and ST8SiaIV inhibit Polysialylation of NCAM in rhabdomyosarcoma tumor cells. BMC Biotechnol..

[28] Bull C., Boltje T.J., Balneger N., Weischer S.M., Wassink M., van Gemst J.J., Bloemendal V.R., Boon L., van der Vlag J., Heise T. (2018). Sialic Acid Blockade Suppresses Tumor Growth by Enhancing T-cell-Mediated Tumor Immunity. Cancer Res..

[29] Wang X., Guo G., Guan H., Yu Y., Lu J., Yu J. (2019). Challenges and potential of PD-1/PD-L1 checkpoint blockade immunotherapy for glioblastoma. J. Exp. Clin. Cancer Res..

[30] Mathieu S., Prorok M., Benoliel A.M., Uch R., Langlet C., Bongrand P., Gerolami R., El-Battari A. (2004). Transgene expression of alpha(1,2)-fucosyltransferase-I (FUT1) in tumor cells selectively inhibits sialyl-Lewis x expression and binding to E-selectin without affecting synthesis of sialyl-Lewis a or binding to P-selectin. Am. J. Pathol..

[31] Souberan A., Cappai J., Chocry M., Nuccio C., Raujol J., Colin C., Lafitte D., Kovacic H., Quillien V., Baeza-Kallee N. (2019). Inhibitor of Apoptosis Proteins Determines Glioblastoma Stem-Like Cell Fate in an Oxygen-Dependent Manner. Stem Cells.

[32] Denicolai E., Baeza-Kallee N., Tchoghandjian A., Carre M., Colin C., Jiglaire C.J., Mercurio S., Beclin C., Figarella-Branger D. (2014). Proscillaridin A is cytotoxic for glioblastoma cell lines and controls tumor xenograft growth in vivo. Oncotarget.

[33] Correard F., Roy M., Terrasson V., Braguer D., Esteve M.A., Gingras M. (2018). Delaying Anticancer Drug Delivery by Self-Assembly and Branching Effects of Minimalist Dendron-Drug Conjugates. Chemistry.

[34] Tchoghandjian A., Souberan A., Tabouret E., Colin C., Denicolai E., Jiguet-Jiglaire C., El-Battari A., Villard C., Baeza-Kallee N., Figarella-Branger D. (2016). Inhibitor of apoptosis protein expression in glioblastomas and their in vitro and in vivo targeting by SMAC mimetic GDC-0152. Cell Death Dis..

[35] Pfaffl M.W. (2001). A new mathematical model for relative quantification in real-time RT-PCR. Nucleic Acids Res..

