# Supplementary material for: Glycolipids Recognized by A2B5 Antibody Promote Proliferation, Migration, and Clonogenicity in Glioblastoma Cells
Source: Cancers (Basel). 2019 Aug 28;11(9):1267. doi: 10.3390/cancers11091267 (PMC6769647; doi:10.3390/cancers11091267)
Supplement: Supplementary file 1 [file cancers-11-01267-s001.pdf]

cancers

Supplementary Materials: Glycolipids Recognized by A2B5 Antibody Promote Proliferation, Migration, and Clonogenicity in Glioblastoma Cells

Nathalie Baeza-Kallee, Raphaël Bergès, Aurélie Soubéran, Carole Colin, Emilie Denicolaï, Romain Appay, Aurélie Tchoghandjian and Dominique Figarella-Branger

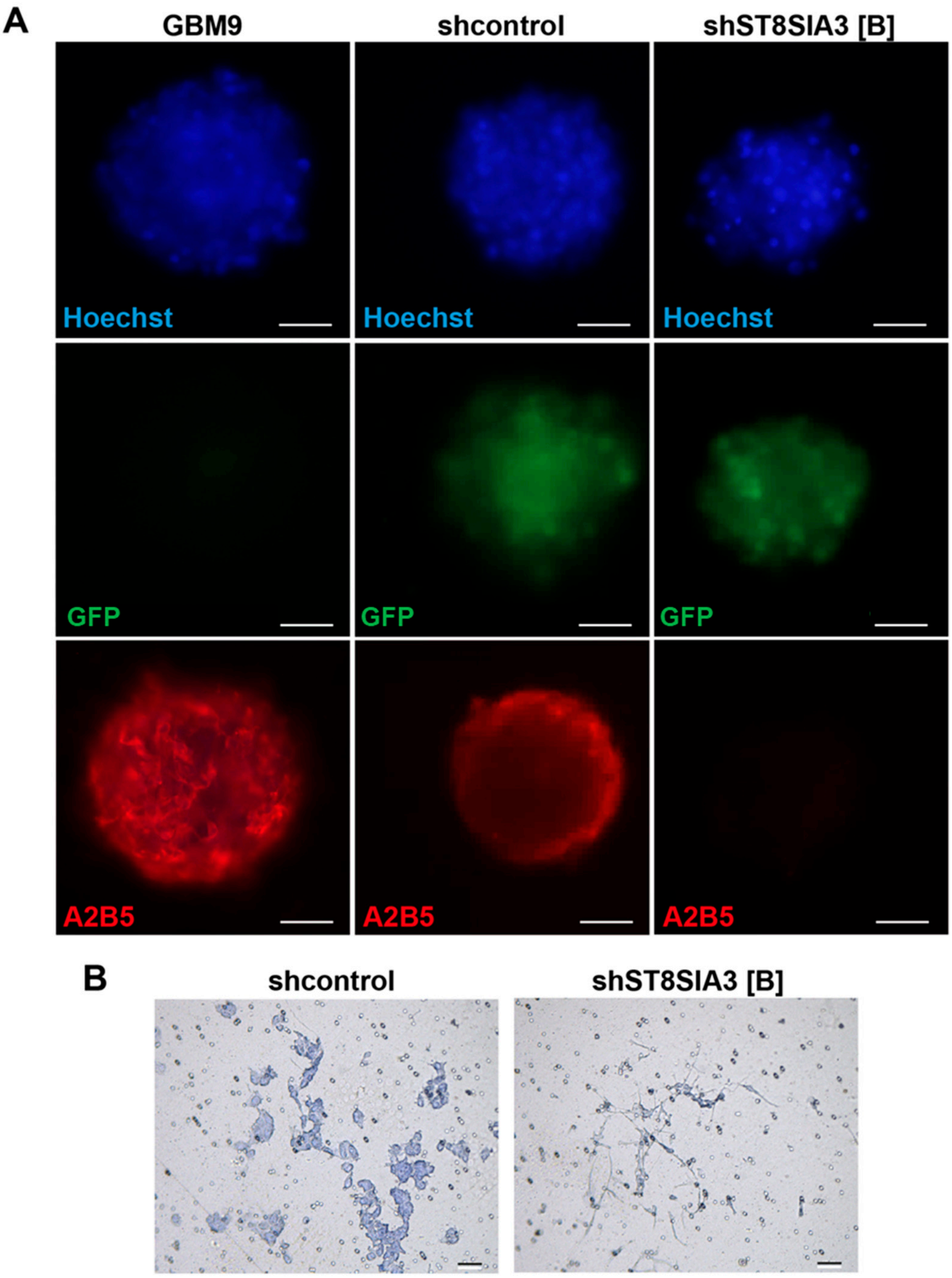

Figure S1. ST8SIA3 silencing in high-A2B5-expressing cells leads to cell death. (**A**) Native GBM9, GBM9-shcontrol, and GBM9-shST8SIA3 [B] secondary spheres were immunolabelled with A2B5 (red). Cell nuclei are counterstained in Hoechst (blue). GFP visualization is consistent with

pGFP-C-shLenti vector lentiviral infection (green). Scale bar = 20 μm. (**B**) Representative phase contrast images of GBM9-shcontrol and GBM9-shST8SIA3 [B] migrating cells (one experiment). Scale bar = 50 μm.

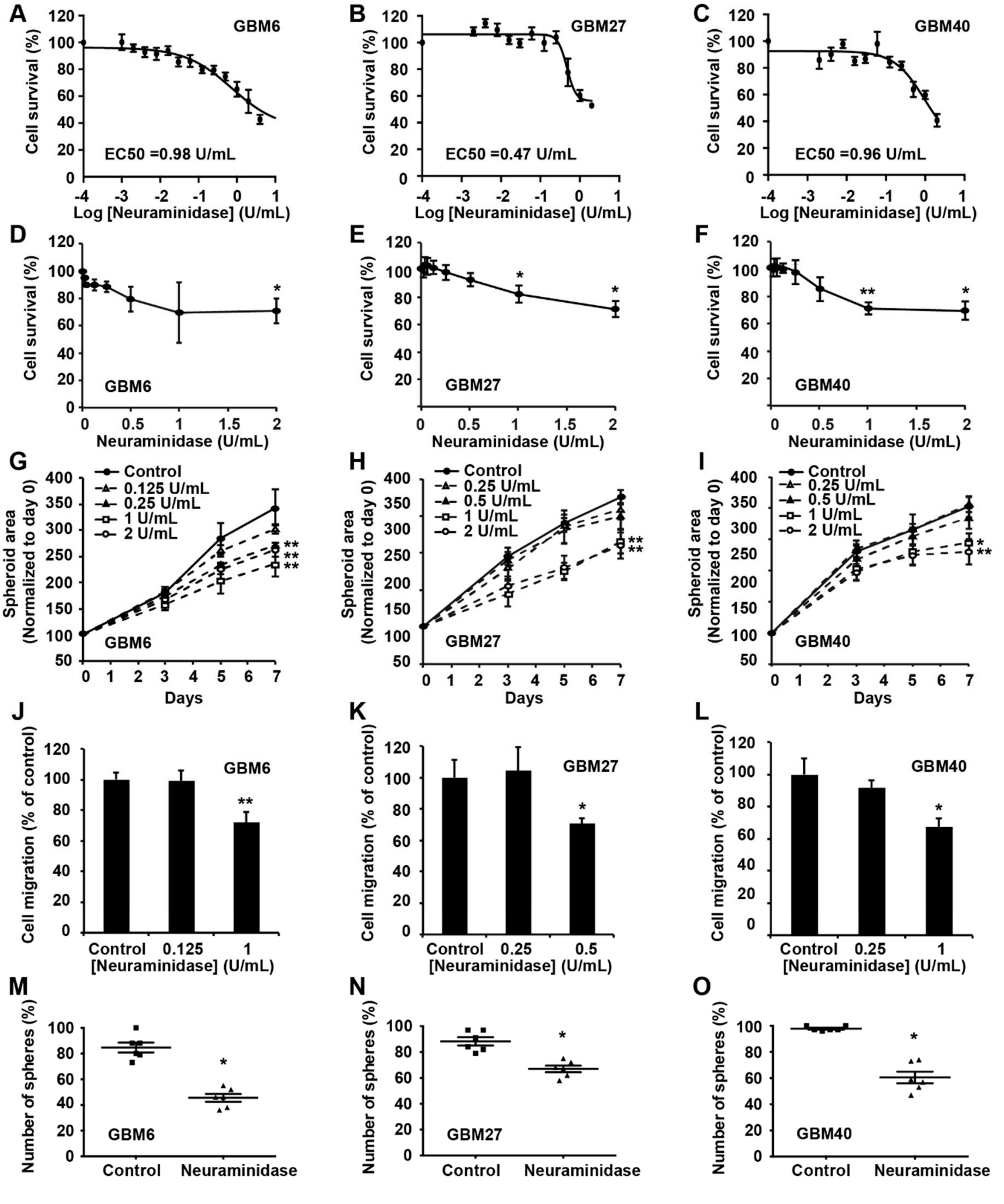

Figure S2. Suppression of A2B5 immunoreactivity in GBM6, GBM27, and GBM40 by neuraminidase impairs cell proliferation, migration, and clonogenicity (**A**)(**B**)(**C**) Dose–response cytotoxicity assays of neuraminidase were conducted for 72 h to assess the viability of GBM6, GBM27, and GBM40 cells grown as a 2D monolayer ($n = 3$). (**D**)(**E**)(**F**) Dose–response cytotoxicity assays of neuraminidase were conducted for 7 days to assess the viability of GBM6, GBM27, and GBM40 cells grown as 3D spheroids ($n = 3$). (**G**)(**H**)(**I**) GBM6, GBM27, and GBM40 spheroid areas were measured during the 7 days after neuraminidase treatment and normalized to Day 0. (**J**)(**K**)(**L**) Quantification of GBM6, GBM27, and GBM40 cell migration using the transwell assay after neuraminidase treatment at 0.125, 0.25, 0.5, or 1 U/mL for 6 h. The mean + SEM values of four independent experiments, each

performed in duplicate, are shown. (**M**)(**N**)(**O**) The self-renewal capacity of GBM6, GBM27, and GBM40 was evaluated by limiting dilution assay in 96-well plates. The point cloud shows the percentage of spheres formed after 8 days of culture of cells treated or not with the respective EC50 neuraminidase dose. Non-parametric Wilcoxon-Mann-Whitney test shows * $p < 0.05$, ** $p < 0.01$.

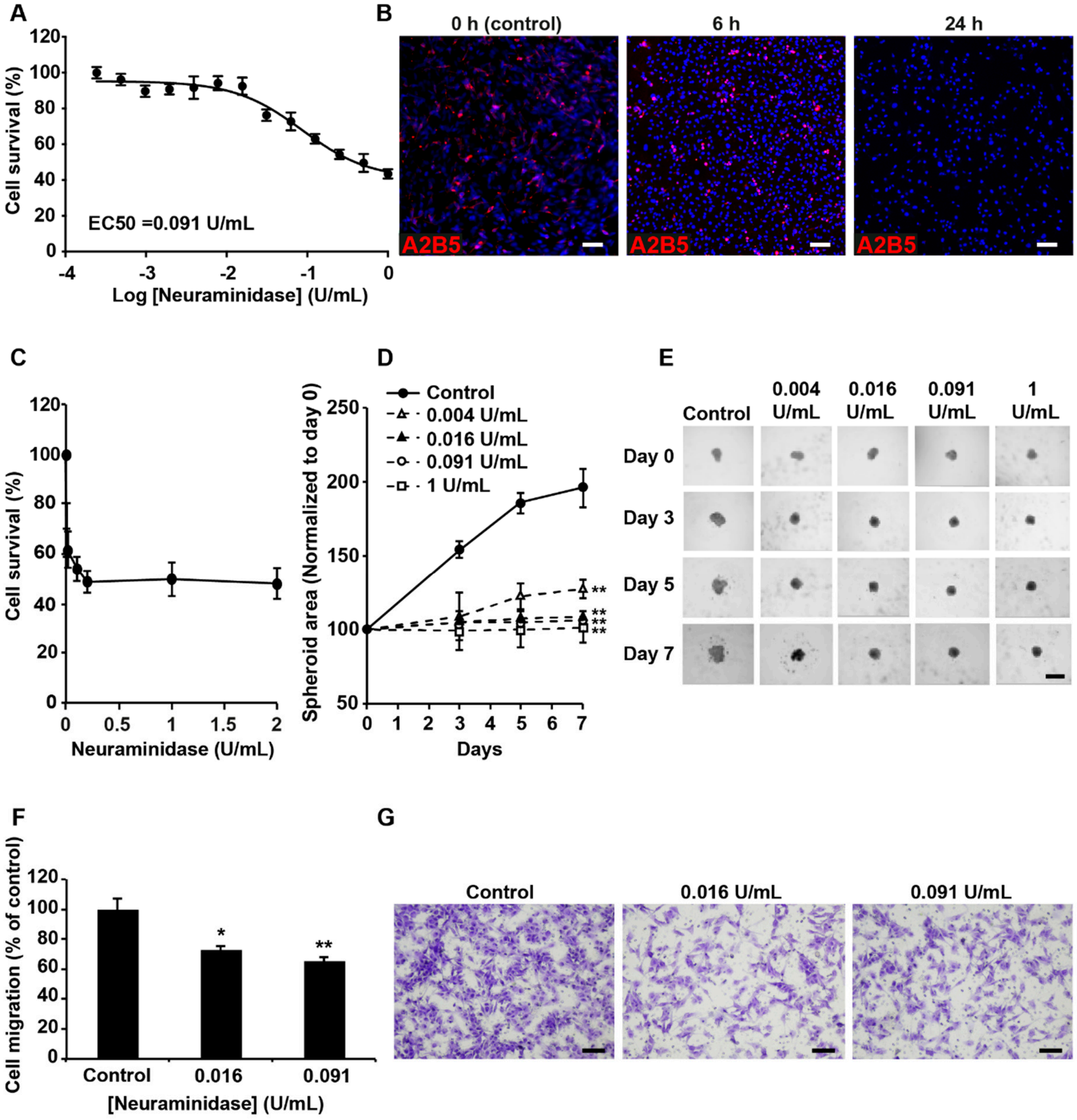

Figure S3. Suppression of A2B5 immunoreactivity in U251-MG cells by neuraminidase induces a decrease in cell proliferation, migration, and clonogenicity (**A**) A dose-response cytotoxicity assay of neuraminidase was conducted for 72 h to assess the viability of U251-MG cells grown as a 2D monolayer ($n = 3$). (**B**) A2B5 staining (in red) on U251-MG control or cells treated with 0.091 U/mL neuraminidase (EC50) for 6 and 24 h. (**C**) A dose–response cytotoxicity assay of neuraminidase was conducted for 7 days to assess the viability of U251-MG cells grown as 3D spheroids ($n = 3$). (**D**) The U251-MG spheroid area was measured during the 7 days after neuraminidase treatment and normalized to Day 0. (**E**) Representative phase contrast images at days 0, 3, 5, and 7 of control and treated U251-MG spheroids. (**F**) Quantification of U251-MG cell migration using the transwell assay after neuraminidase treatment at 0.016 and 0.091 U/mL (EC50) for 6 h. The mean + SEM values of four independent experiments, each performed in duplicate, are shown. (**G**) Representative phase contrast images of control and treated migrating U251-MG cells. Scale bar = 50 µm. Non-parametric Wilcoxon-Mann-Whitney test shows * $p < 0.05$, ** $p < 0.01$.

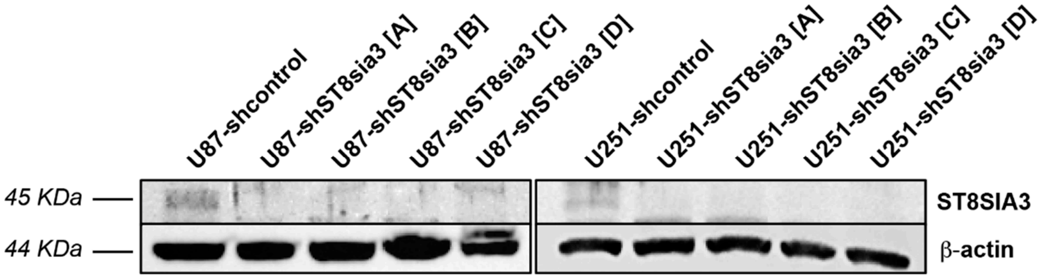

Figure S4. Western blot analysis of ST8SIA3 in shST8SIA3 cell clones. Western blot analysis of ST8SIA3 protein in U87-shcontrol; U87-shST8SIA3 [A], [B], [C], and [D]; U251-shcontrol; and U251-shST8SIA3 [A], [B], [C], and [D] grown as a subconfluent monolayer in DMEM with 10% FCS. The expression level of β-actin served as a loading control.

Figure S5. Time-lapse of the migration of GBM explants over 24 h, untreated (Video 1) or treated with neuraminidase (1 U/mL) (Video 2). Acquisitions were made every 10 min over 24 h of recording (videos: 30 frames per second). Scale bar = 200 µm. Video 1 shows the migration of the explant's cells and, therefore, a spreading of the explant. Video 2 shows that neuraminidase induces retraction of the explant on itself at early treatment times.

© 2019 by the authors. Submitted for possible open access publication under the terms and conditions of the Creative Commons Attribution (CC BY) license (http://creativecommons.org/licenses/by/4.0/).
